# Massive MIMO Systems for 5G and beyond Networks—Overview, Recent Trends, Challenges, and Future Research Direction

**DOI:** 10.3390/s20102753

**Published:** 2020-05-12

**Authors:** Robin Chataut, Robert Akl

**Affiliations:** Department of Computer Science and Engineering, University of North Texas, Denton, TX 76203, USA; Robert.Akl@unt.edu

**Keywords:** 5G, 6G, beamforming, channel estimation, massive MIMO, millimeter waves, pilot contamination, signal detection, spectral efficiency, terahertz spectrum

## Abstract

The global bandwidth shortage in the wireless communication sector has motivated the study and exploration of wireless access technology known as massive Multiple-Input Multiple-Output (MIMO). Massive MIMO is one of the key enabling technology for next-generation networks, which groups together antennas at both transmitter and the receiver to provide high spectral and energy efficiency using relatively simple processing. Obtaining a better understating of the massive MIMO system to overcome the fundamental issues of this technology is vital for the successful deployment of 5G—and beyond—networks to realize various applications of the intelligent sensing system. In this paper, we present a comprehensive overview of the key enabling technologies required for 5G and 6G networks, highlighting the massive MIMO systems. We discuss all the fundamental challenges related to pilot contamination, channel estimation, precoding, user scheduling, energy efficiency, and signal detection in a massive MIMO system and discuss some state-of-the-art mitigation techniques. We outline recent trends such as terahertz communication, ultra massive MIMO (UM-MIMO), visible light communication (VLC), machine learning, and deep learning for massive MIMO systems. Additionally, we discuss crucial open research issues that direct future research in massive MIMO systems for 5G and beyond networks.

## 1. Introduction

**Figure 1:**
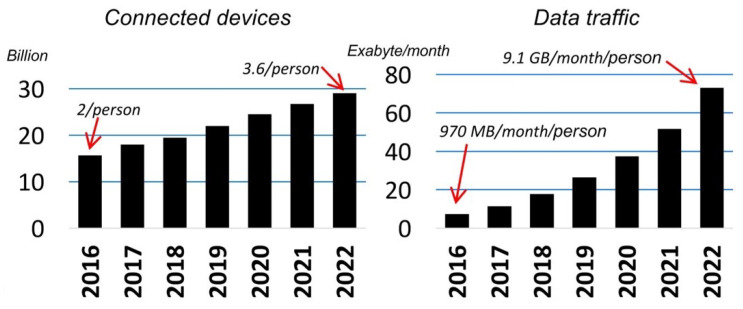
Global mobile data traffic and growth in connected devices from 2017 to 2022.

With globalization, present-day networks are facing high traffic demands, and to fulfill these needs, cellular systems are deployed within a few hundred-meter distances, and wireless Local Area Networks (LAN) are placed almost everywhere. Along with increased mobile broadband service, the introduction of new concepts like the Internet of Things (IoT) and Machine-to-Machine Communication (M2M) are also contributing to the increased wireless traffic. The global deployment of cellular service cultivates the cell phone users to be used to the mobile data in their day to day life tremendously. The services like video calling, online gaming, social media applications like Facebook, Twitter, WhatsApp, have changed our life drastically with the capabilities of the third-generation (3G), fourth-generation (4G), and fifth-generation (5G) networks, like lower latency and high data rate [1]. A full cell phone connected world is expected in the next few years, which will be mainly characterized by growth in users, connectivity, data traffic volume, and a wide range of applications. In the next few years, technology like augmented reality, virtual reality, ultra high definition video, 3D video, and features like a mobile cloud will become popular to enrich the ultimate user experience. From 2017–2022, smartphone traffic is expected to increase by ten times, and overall, mobile traffic will be increased by eight times [2]. Figure 1 shows the growth in mobile data traffic and the number of connected devices from 2017–2022 [3]. By the end of 2022, more than 90 percent of the traffic will come from cell phones. This colossal amount of mobile data traffic is challenging to manage with the capabilities of previous wireless generation systems.

The primary issue with the ongoing development of the wireless network is that it is dependent upon either increasing bandwidth (spectrum) or densifying the cells to achieve the required area throughput. These resources are rare and are reaching their saturation point within a few years. Also, increasing bandwidth or densifying the cells increases the cost of the hardware and increases latency. The third factor, which can improve area throughput, that is, spectral efficiency, has remained mostly untouched and unchanged during this rapid development and growth of the wireless network. An efficient wireless access technology that can increase the wireless area throughput without increasing the bandwidth or densifying the cell is essential to achieve the ongoing demands faced by the wireless carriers.

Massive Multiple-Input Multiple-Output (MIMO) is the most enthralling wireless access technology to deliver the needs of 5G and beyond networks. Massive MIMO is an extension of MIMO technology, which involves using hundreds and even thousands of antennas attached to a base station to improve spectral efficiency and throughput. This technology is about bringing together antennas, radios, and spectrum together to enable higher capacity and speed for the incoming 5G [4,5]. The capacity of massive MIMO to increase throughput and spectral efficiency has made it a crucial technology for emerging wireless standards [6,7]. The key here is the considerable array gain that massive MIMO achieves with a large number of antennas [8]. Massive MIMO is a key enabling technology for 5G and beyond networks, and as intelligent sensing system primarily rely on 5G and beyond networks to function, massive MIMO and intelligent sensing system are inextricably linked. The data collection from the large number of smart sensors using traditional multi-access schemes is very impractical as it leads to excessive latency, low data rate, and reduced reliability. Massive MIMO with huge multiplexing gain and beamforming capabilities can sense data from concurrent sensor transmission with much lower latency and provide sensors with higher data rates and reliable connectivity. Massive MIMO systems will perform a crucial role to allow information gathered through smart sensors to be transmitted in real-time to central monitoring locations for smart sensor applications such as an autonomous vehicle, remote healthcare, smart grids, smart antennas, smart highways, smart building, and smart environmental monitoring.

The rest of the paper is organized as follows: Section 2 provides details on the evolution of cellular networks from the first-generation (1G) to sixth-generation (6G) networks. Section 3 provides insights into key enabling technologies for 5G networks. The benefits of massive MIMO are explained in Section 4, and Section 5 provides a brief description of the importance of massive MIMO for future generation networks. Section 6 reviews the challenges in massive MIMO systems and explains some state-of-the-art mitigation techniques. Section 7 discusses the possibility of our current phone to use the massive MIMO technology, and Section 8 presents the use of machine learning and deep learning in massive MIMO systems. Section 9 presents the active research topic on massive MIMO systems for future generation networks, and Section 10 concludes the paper summarizing the key ideas.

## 2. Evolution of Cellular Networks

**Figure 2:**
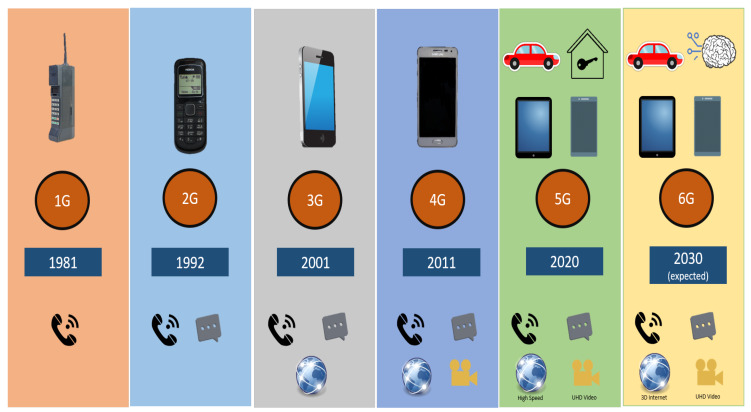
The evolution of mobile communication from 1G to 5G.

The mobile communication era started in the early 1980s, and since then, mobile communication has experienced tremendous growth in the past few decades. Cellular networks have evolved from 1G to 5G and beyond. All cellular networks are composed of base stations, user equipment (phones), and core networks. The evolution from 1G to 6G is summarized in Figure 2.

### 2.1. 1G

The 1G mobile networks were introduced in the early 1980s and used analog signals for voice-only services. 1G systems used Frequency Division Multiple Access (FDMA) and offered data rates up to 2.4 kbps. They had poor voice quality due to high interference. 1G systems included Advanced Mobile Phone Systems (AMPS), Total Access Communication System (TACS), and Nordic Communication System (NMTS) [4].

### 2.2. 2G

The second-generation (2G) mobile networks were introduced in the early 1990s and were generally considered digital versions of 1G networks. Along with voice services, they allowed Short Message Service (SMS) and basic email services. These systems used Code Division Multiple Access (CDMA) and Time Division Multiple Access (TDMA) and offered data rates from 14.4 kbps up to 64 kbps. 2G systems included Global System for Mobile Communication (GSM) and IS-95 CDMA. 2G networks have limited mobility and hardware capability [4].

### 2.3. 2.5G and 2.75G

2G technology was continuously improving to provide better data rates and services, and thus 2.5G networks were introduced with data rates up to 384 kbps. 2.5G systems included General Packet Radio Service (GPRS), Enhanced Data GSM Evolution (EDGE), and CDMA2000.

### 2.4. 3G

The 3G mobile networks were introduced in the early 2000s and were based on GSM and CDMA. These systems offered web browsing on mobile phones along with voice, Multimedia Message Support (MMS), and SMS services. 3G systems included Universal Mobile Telecommunication Systems (UMTS) and WCDMA. Smartphones became popular in the mid-2000s. 3G networks provided data rates upward of 384 Kbps, but they required large bandwidth and complex infrastructure.

### 2.5. 3.5G

Due to continuous demand for higher data rates, High-Speed Downlink Packet Access (HSDPA), High-Speed Uplink Packet Access (HSUPA), and High-Speed Packet Access (HSPA+) were introduced in 3G networks to increase data rates. These types of networks were referred to as 3.5G networks, and they provided data rates up to 2 Mbps. Although 3.5G provided a higher data rate, the implementation and the equipment was costly, and compatibility with 2G was very challenging [4].

### 2.6. 4G

The 4G mobile networks were introduced in the early 2010s. 4G networks offer data rates up to 100 Mbps and can handle more data traffic with a better quality of service (QoS). 4G networks include applications like video conferencing, online gaming, and mobile television. 4G systems include Worldwide Interoperability for Microwave Access (WiMAX), Long Term Evolution (LTE), and LTE-Advanced (LTE-A), and it has feasible compatibility with older generation networks [9]. The frequency bands of 4G are considerably expensive, and high-end 4G enabled cell phones are required to operate 4G networks [9].

### 2.7. 5G

**Figure 3:**
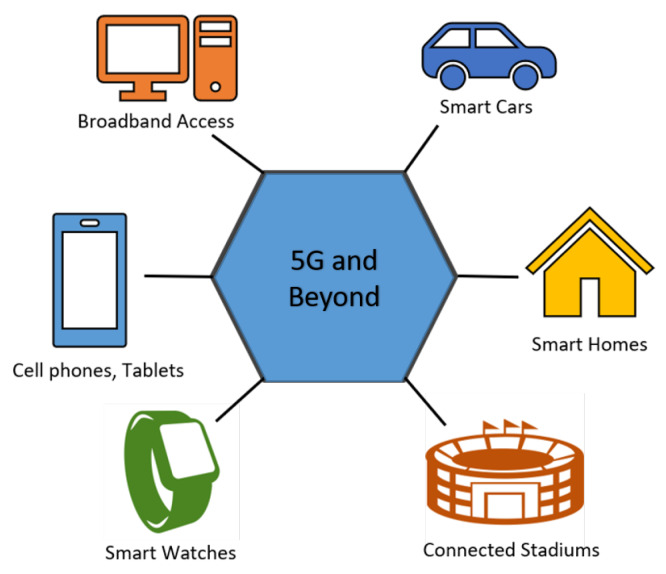
Factors contributing to more increment in wireless data traffic.

The 5G mobile networks are currently starting to be implemented and aim to be 100 times faster than current 4G networks. 5G networks will offer data rates up to 10 Gbps, low latency (in milliseconds), and greater reliability. Imagine that an HD movie can be downloaded in just a few seconds. This technology can support many Internet of Things (IoT) enabled devices and smart vehicles, as shown in Figure 3. Efficient wireless access technology that can increase throughput without increasing the bandwidth or densifying the cell is essential to achieve the ongoing demands faced by 5G. Some of the significant advantages of 5G are:

- **Data rate:** 5G network would provide data rate up to 10 Gbps, which is almost a hundred times better than 4G networks.
- **Latency:** 5G network provides latency as low as 1 ms compared to 10 ms latency provided by 4G networks.
- **Efficient signaling:** 5G networks provide efficient signaling for IoT connectivity and M2M communication.
- **User experience:** 5G enhances augmented reality, virtual reality, and artificial intelligence.
- **Spectral efficiency:** 5G would provide ten times more spectral and network efficiency compared to 4G networks.
- **Energy efficiency:** 5G networks provide 90 % more efficient network energy usage compared to 4G networks.
- **Ubiquitous Connection:** 5G provides huge broadcasting data, which can support more than 65,000 connections, which is a hundred times more than 4G networks.
- **Battery life:** 5G provides almost ten years of battery life for low powered IoT devices.

Along with immense advantages, 5G technology comes with certain challenges. Some of the challenges for 5G technology are:

- **Frequency bands:** Frequency bands up to 300 GHz have been considered for 5G networks. These high-frequency bands are costly, and wireless carriers will have to pay millions to get this high-frequency spectrum.
- **Coverage:** The high-frequency wave has a shorter wavelength; thus, it cannot travel to a longer distance. Due to this issue, there should be more base stations in a smaller area to give each user a reliable connection. The additional base station increases the cost and complexity of the overall network.
- **Cost:** Since 5G is not just about adding an extra layer to the 4G network, the cost to build the system from the base level is prohibitive.
- **Device Support:** Since the phones available in the current market does not support 5G infrastructure, and it would be a challenge for device manufacturers to develop cheaper phone which can support 5G.
- **Security and Privacy:** Although 5G uses the authentication and Key Agreement (AKA) system, it is still venerable from attacks such as middle man attack, location tracking, and eavesdropping.
- **Availability:** With the introduction of M2M and IoT, network overload and congestion would be a major problem in the future. These radio access network challenges will make it difficult to make the network available to everyone.
- **Cybercrime:** With high speed, data Cybercrime would increase drastically. Thus, strict Cyberlaws would be necessary to prevent these attacks.

### 2.8. 6G

The 6G mobile networks are complete wireless networks with no limitation. It is currently in the developmental stage, and it will provide incredible transmission speed in the terabit range. This technology would require a smart antenna, large memory in cell phones, and huge optical networks. The 6G networks will be cell-free, and it would enable artificial intelligence in wireless networks. It is not clear what frequency band 6G networks will use, but it is apparent that a much higher frequency band will be needed to increase the data rate required for 6G networks. While 5G is supposed to use a frequency greater than 30 GHz and up to 300 GHz (millimeter waves), 6G is associated with much higher frequency in THz bands (300 GHz to 3 THz). The use of the THz spectrum for 6G is estimated to become commercial is the next 5–7 years. Some of the applications for 6G networks are connected robotics and autonomous systems, wireless brain-computer interfaces, blockchain technology, multi-sensory extended reality, space travel, deep-sea sightseeing, tactile internet, and industrial internet. 6G networks are expected to be introduced in the year 2030. Some of the advantages of 6G networks are:

- **Data rate:** 6G network is expected to provide data rate up to 10 Tbps, which is almost a hundred times better than 5G networks.
- **Latency:** 6G network would provide latency as low as 0.1 ms compared to 1 ms latency provided by 5G networks.
- **Efficient signaling:** 6G networks provide efficient signaling for massive IoT connectivity and M2M communication.
- **User experience:** 6G enhances extended reality, augmented reality, virtual reality, and artificial intelligence.
- **Spectral efficiency:** 6G would provide ten times more spectral and network efficiency compared to 5G networks.
- **Energy efficiency:** 6G networks provide 100 times more efficient network energy usage compared to 5G networks.
- **Ubiquitous Connection:** 6G will provide huge broadcasting data, which can support more than 1 million connections, which is almost a hundred times more than 5G networks.

Table 1 shows the feature comparison of 4G, 5G, and 6G networks.

## 3. Key Enabling Technologies for 5G and Beyond Networks

**Figure 4:**
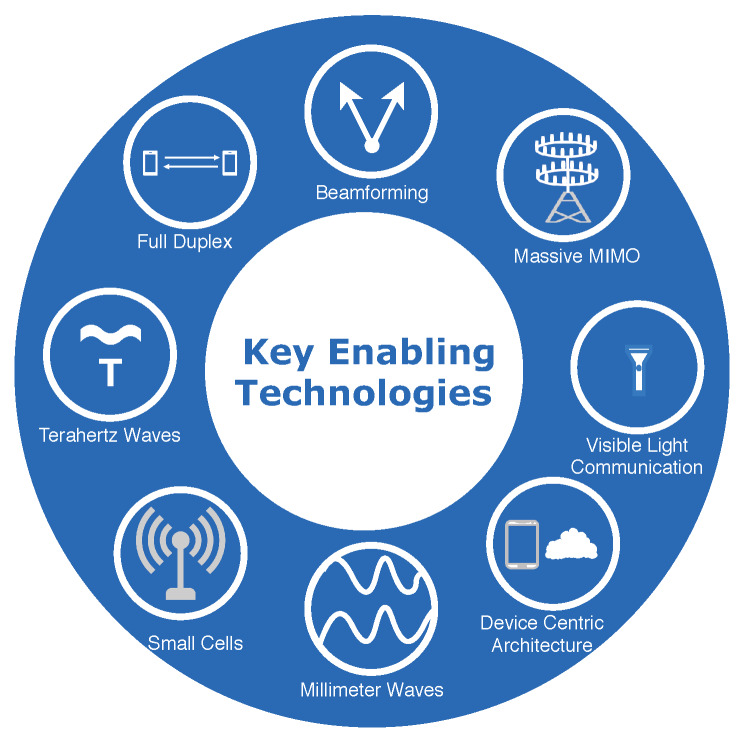
The 8 Key enabling technologies for 5G and beyond networks.

To make 5G and beyond networks a reality, many advanced ideas have been proposed and analyzed in recent years. The major key enabling technologies that have been considered for 5G and 6G systems include millimeter waves, small cells, beamforming, device-centric architecture, full-duplex technology, massive MIMO, Terahertz wave, and visible light spectrum as shown in Figure 4.

### 3.1. Millimeter Waves

Generally, a frequency below 6 GHz is used for cellular communication, and frequency above that is mostly used for other services like medical imaging, microwave remote sensing, amateur radio, terahertz computing, and radio astronomy. The massive increase in data traffic has made the radio frequency spectrum congested. The result is that there is limited bandwidth for a user, causing a slower and unreliable connection. One way to solve this problem is by using frequency above 6 GHz for wireless communication. The frequency above 6 GHz has never been used for wireless communication, and there has been a lot of research going on with broadcasting millimeter waves. Millimeter waves are frequency between 30 GHz to 300 GHz, and it is called millimeter waves because its length varies from 1 to 10 mm compared to the radio waves that are used in the current mobile communication system, which measure tens of centimeters in length.

Many aspects of millimeter waves are published in the past few years [10,11]. Authors in [12,13] discuss the potentials and challenges in the millimeter-wave technology. The future of the 5G network with millimeter wave technology is presented in [14]. Millimeter waves can provide bandwidth ten times more than that of the entire 4G cellular band. These high-frequency waves are used in some satellite application, but it has never been used for mobile broadband. Since millimeter has a lower wavelength, they are not suitable for long-range applications. Another problem with millimeter waves is that they cannot penetrate buildings and obstacles, and they tend to get absorbed by rain.

### 3.2. Sub-Millimeter or Terahertz Band

With globalization, the current wireless market is expanding rapidly. With talk of 6G networks, the demand for a higher spectrum is imminent in the near future. The frequency higher than the millimeter-wave band (30 GHz–300 GHz) could be used for wireless communication. The frequency band between 300 GHz to 3 THz is known as the Terahertz band. Although this idea is relatively new, research in this area can be worthwhile for the wireless communication industry. Other than just a higher spectrum, there are many advantages of THz band, such as interference friendly deployment, scalability, enhanced security, availability of greenfield spectrum, low power consumption, a front-haul boost for the wireless network, small antennas size, and focused beams [15].

**Figure 5:**
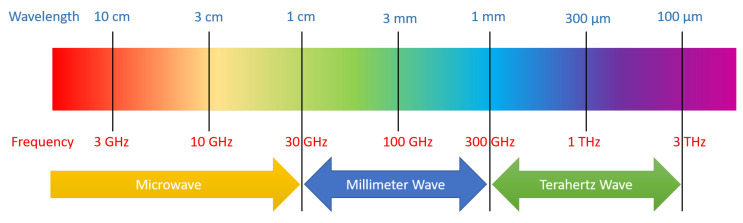
Millimeter and terahertz wave band.

THz technology would be beneficial for applications such as imaging, spectroscopy, holographic telepresence, industry 4.0, and massive scale communications. There are several challenges and new areas of research in THz band deployments such as complex antenna design to support higher antenna gain, access point specification and deployment, complex circuit design, high propagation loss, and complex mobility management [15]. The millimeter-wave and terahertz wave bands are shown in Figure 5.

The concept of ultra massive MIMO (UM-MIMO) has emerged in recent years, which takes advantage of plasmonic materials for building antennas and transceivers to achieve the capacity of THz band. Materials such as graphene and metamaterials can be used to build nano antennas and transceivers. These nano antennas and transceivers can operate in the THz band [16]. UM-MIMO can take advantage of these miniature antennas and transceivers to provide higher spatial multiplexing and beamforming. Thus, the data rates and communication range can be improved with the help of spatial multiplexing and beamforming, respectively. A lot of investigation is needed to realize THz UM-MIMO for 5G and beyond networks. Some of the challenges are the fabrication of plasmonic nano array antennas, channel estimation, precoding, signal detection, beamforming, and beemsteering [16,17].

### 3.3. Small Cells or Heterogeneous Networks

Small cells are low power tiny base stations that can be placed within every 100 m distance to cover small geographical areas. These low power base stations prevent the signal from dropping in crowded areas. Small cells are very light and small; thus, they can be placed anywhere. If we are using millimeter waves instead of the traditional sub-6 GHz spectrum, the small cell can become even smaller and can be fitted in tiny places. The small cells will play a significant role in delivering high-speed mobile broadband and ultra-low latency for 5G. Small Cells can be further divided into microcells, femtocells, and picocells based on coverage area and the number of users it can support. Several studies of smalls cells and its benefits for 5G networks are studied in [18].

### 3.4. Beamforming

**Figure 6:**
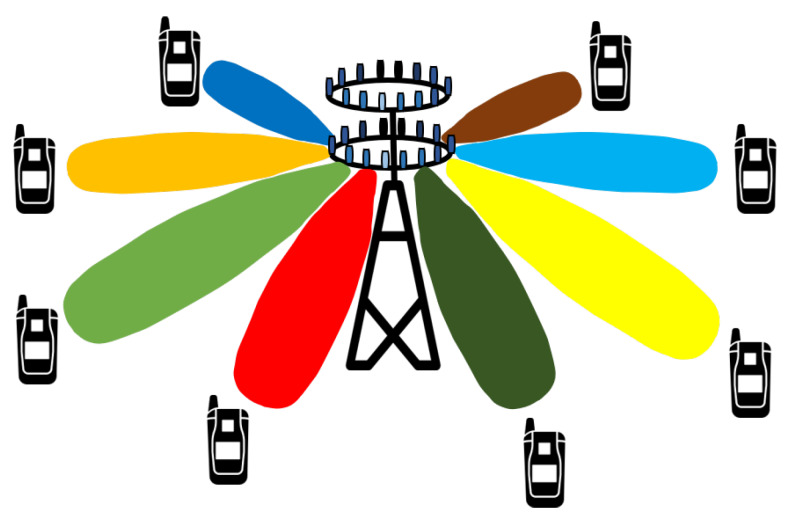
Massive Multiple Output–Multiple Output (MIMO) beamforming.

Beamforming is the ability of the base station to adapt the radiation pattern of the antenna [19]. Beamforming helps the base station to find a suitable route to deliver data to the user, and it also reduces interference with nearby users along the route [20], as shown in Figure 6. Beamforming has several advantages for 5G networks and beyond. Depending upon the situation, beamforming technology can be implemented in several different ways in future networks. For massive MIMO systems, beamforming helps with increasing spectrum efficiency, and for millimeter waves, it helps in boosting data rate. In massive MIMO systems, the base station can send data to the user from various paths, and beamforming here choreographs the packet movement and arrival time to allow more users to send data simultaneously. Since the millimeter waves cannot penetrate through obstacles and do not propagate to longer distances due to a shorter wavelength, beamforming here helps to send concentrated beams towards the users. Thus, beamforming helps a user to receive a strong signal without interference with other users.

### 3.5. Device Centric Architecture

The current 4G system relies on base station centric architecture where a device relies on downlink and uplink connection and control and data channel to obtain the services from the base station. With an increased number of users, cell density or base station density is increasing rapidly, and this densification in the network would require major changes in the 5G and beyond networks. Also, with the introduction of millimeter waves, many frequency bands with entirely different propagation characteristics will coexist together. Thus a base station centric architecture might evolve into a device-centric architecture in future networks to overcome challenges like network densification and increased frequency bands [21].

**Figure 7:**
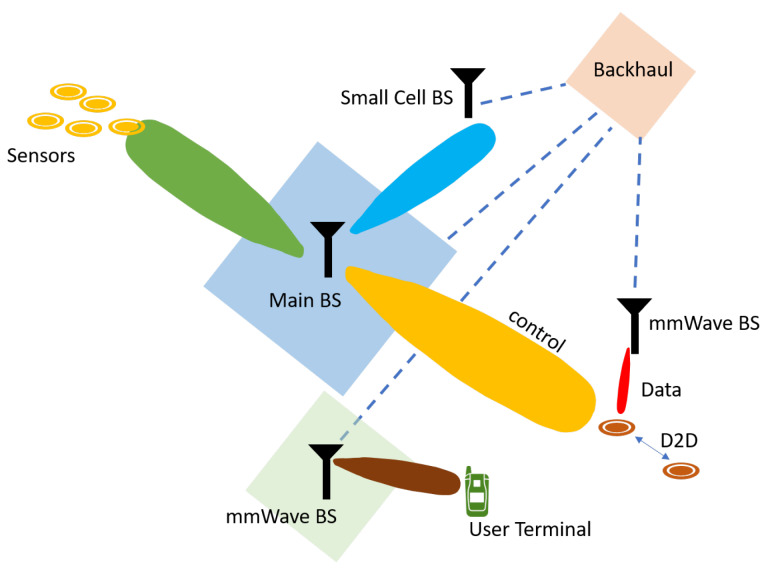
Device centric architecture.

In device-centric architecture, a user device would communicate by exchanging information through several heterogeneous nodes [22]. Various research on the benefits of device-centric architecture for 5G networks is presented in Reference [23]. A typical device-centric architecture is shown in Figure 7.

### 3.6. Full Duplex Technology

**Figure 8:**
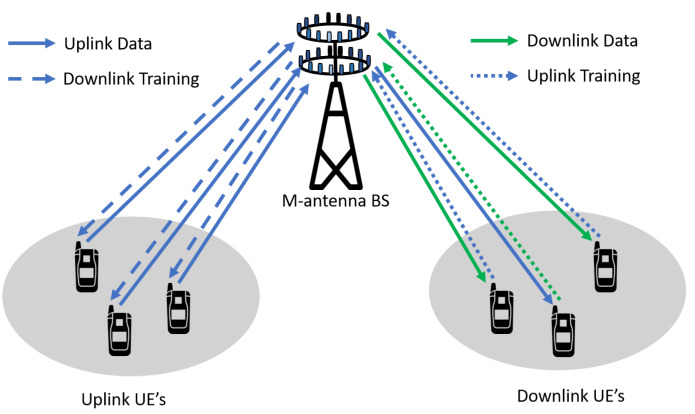
Full duplex technology.

Generally, wireless transmission and reception are not done at the same frequency bands to avoid interference. Any bidirectional system thus has to separate the uplink and downlink channel using time or frequency domain to get orthogonal non-interfering signals. Full duplex refers to the simultaneous transmission and reception over the same frequency band and at the same time, as shown in Figure 8. 5G networks will use full-duplex for the transmission of signals to potentially double the network capacity and is beneficial for higher layers (e.g., MAC layer). One of the disadvantages of full-duplex technology is that it increases signal interference thought pesky echo [24]. Several studies have been conducted on full-duplex technology and its benefits for 5G networks [25,26].

### 3.7. Visible Light Communication

Visible Light Communication (VLC) provides optical fiber like performance for future generation networks. It uses visible light between 400 and 800 THz using both fluorescent lamps or LEDs to transmit the signal over the shorter distance. VLC can be built with very low-cost hardware, and it can take advantage of the unlicensed band. VLC does not induce any electromagnetic radiation, which makes it unexposed to external electromagnetic radiation. Since this technology requires an illumination source, this technology is mostly useful for indoor applications. A standard for VLC has been defined in IEEE 802.15.7, but 3rd Generation Partnership Project (3GPP) has not considered it for cellular networks [27]. VLC would be very useful for smart city applications, and it has been recognized as one of the key enabling technologies for 6G networks.

### 3.8. Massive MIMO

MIMO systems are an integral part of current wireless systems, and in recent years they have been used extensively to achieve high spectral efficiency and energy efficiency. Before the introduction of MIMO, single-input-single-output systems were mostly used, which had very low throughput and could not support a large number of users with high reliability. To accommodate this massive user demand, various new MIMO technology like single-user MIMO (SU-MIMO) [28,29], multi-user MIMO (MU-MIMO) [30,31,32,33] and network MIMO [34,35] were developed. However, these new technologies are also not enough to accommodate the ever-increasing demands. The wireless users have increased exponentially in the last few years, and these users generate trillions of data that must be handled efficiently with more reliability.

Additionally, there are billions of IoT devices, having various applications to smart health-care, smart homes, and smart energy, that contribute to the data traffic. It is predicted that there will be around 50 billion connected devices by the end of 2020. The current MIMO technologies associated with 4G/LTE network is unable to handle this huge influx in data traffic with more speed and reliability. Thus, the 5G network is considering massive MIMO technology as a potential technology to overcome the problem created by massive data traffic and users [6,36]. Several studies on massive MIMO have been conducted on massive MIMO systems and their benefits [7,37].

**Figure 9:**
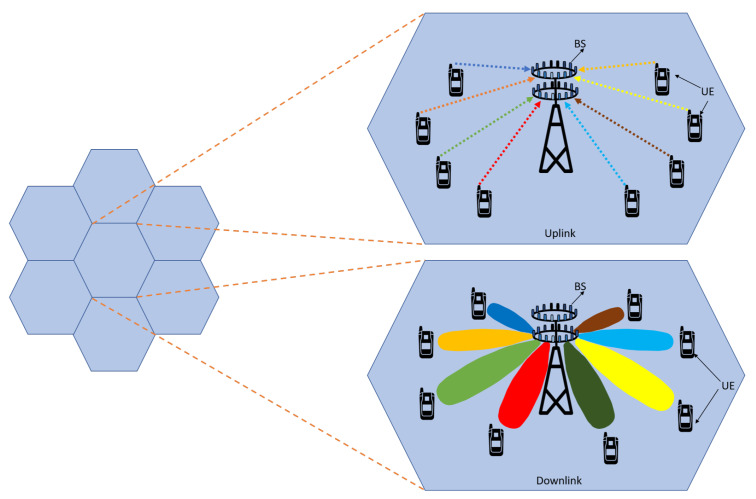
Massive MIMO uplink and downlink.

**Figure 10:**
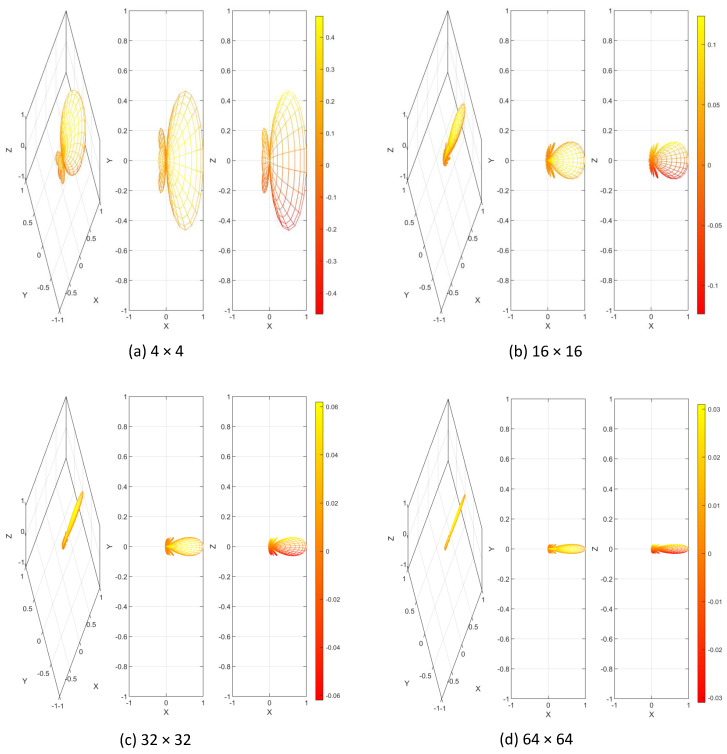
Beam Pattern with different antenna configuration. (**a**) 4 × 4 MIMO (**b**) 16 × 16 MIMO (**c**) 32 × 32 MIMO (**d**) 64 × 64 MIMO.

Massive MIMO is the most captivating technology for 5G and beyond the wireless access era. Massive MIMO is the advancement of contemporary MIMO systems used in current wireless networks, which groups together hundreds and even thousands of antennas at the base station and serves tens of users simultaneously [38,39]. The extra antennas that massive MIMO uses will help focus energy into a smaller region of space to provide better spectral efficiency and throughput. Massive MIMO downlink and the uplink system is shown in Figure 9. As the number of antenna increases in a massive MIMO system, radiated beams become narrower and spatially focused toward the user. The beam patterns for different antenna configurations are shown in Figure 10. These spatially focused antenna beams increase the throughput for the desired user and reduce the interference to the neighboring user [40]. Massive MIMO offers an immense advantage over the traditional MIMO system, which are summarized in Table 2 [41].

#### 3.8.1. Uplink Transmission

**Figure 11:**
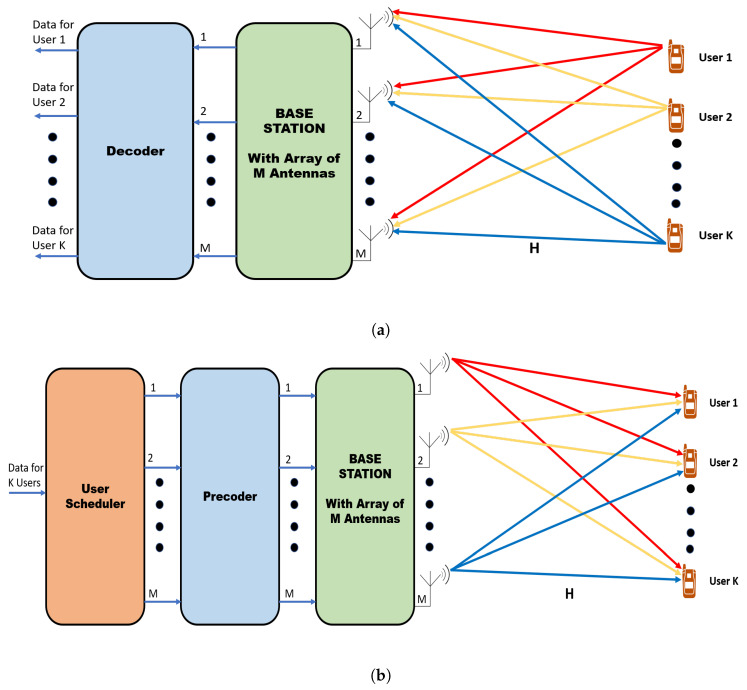
Massive MIMO uplink and downlink operation. (**a**) Uplink (**b**) Downlink.

The uplink channel is used to transmit data and the pilot signal from the user terminal to the base station, as shown in Figure 11a. Let us consider a massive MIMO uplink system equipped with M antennas at the base station and simultaneously communicating with N (M ≫ N) single-antenna users. If the signal transmitted by the user or the deterministic pilot signal to estimate the channel is $x\in C^{N}$, the signal received at the base station during uplink is given as:

(1) $$y=Hx+n_{uplink},$$

where $y\in C^{M}$ is the signal received at the base station, *H* is the channel vector between the user terminal and the base station, and elements of $H\in C^{M\times N}$ are independent and identically distributed with zero mean and unit variance, that is, H∼CN(0,1). The additional term $n_{uplink}\in C^{M}$ is the addition of interference from several transmissions and the receiver noise. The interference added is independent of the user signal *x*, but it can be dependent on the channel *H*.

(2) $$n_{uplink}=n_{uplink-interference}+n_{noise}.$$

#### 3.8.2. Downlink Transmission

The downlink channel is used to transmit data or estimate the channel between user and base station. The base station uses training pilots to estimate the channel. A downlink transmission with several UE and a base station is shown in Figure 11b. Let us consider a downlink massive MIMO system, where base station equipped with *M* antennas, and it is serving *N* users having a single antenna simultaneously. The base station sends independent information to multiple users simultaneously. The signal received, $y_{k}\in C^{M\times 1}$ at the $k_{th}$ user is:

(3) $$y_{k}=h_{k}x_{k}+n_{downlink}.$$

where $h_{k}$ is a channel vector between $k_{th}$ user and base station, whose elements are independent and identically distributed with zero mean and unit variance, that is, h∼CN(0,1). $x_{k}\in C^{M}$ is the signal transmitted by base station for user *k* and, $n_{downlink}$ is the additional noise which is composed of the receiver noise $n_{noise}∼\mathrm{CN}\left(0,\sigma^{2}I\right)$ and the interference during downlink $n_{downlink-interference}$ caused by transmitting simultaneously to other users and is given as:

(4) $$n_{downlink}=n_{downlink-interference}+n_{noise}$$

## 4. Benefits of Massive MIMO for 5G Networks and Beyond

Some of the benefits of massive MIMO technology are:

- **Spectral Efficiency:** Massive MIMO provides higher spectral efficiency by allowing its antenna array to focus narrow beams towards a user. Spectral efficiency more than ten times better than the current MIMO system used for 4G/LTE can be achieved.
- **Energy Efficiency:** As antenna array is focused in a small specific section, it requires less radiated power and reduces the energy requirement in massive MIMO systems.
- **High Data Rate:** The array gain and spatial multiplexing provided by massive MIMO increases the data rate and capacity of wireless systems.
- **User Tracking:** Since massive MIMO uses narrow signal beams towards the user; user tracking becomes more reliable and accurate.
- **Low Power Consumption:** Massive MIMO is built with ultra lower power linear amplifiers, which eliminates the use of bulky electronic equipment in the system. This power consumption can be considerably reduced.
- **Less Fading:** A Large number of the antenna at the receiver makes massive MIMO resilient against fading [42].
- **Low Latency:** Massive MIMO reduces the latency on the air interface [43].
- **Robustness:** Massive MIMO systems are robust against unintended interference and internal Jamming. Also, these systems are robust to one or a few antenna failures due to large antennas [44].
- **Reliability**: A large number of antennas in massive MIMO provides more diversity gain, which increases the link reliability [45,46].
- **Enhanced Security:** Massive MIMO provides more physical security due to the orthogonal mobile station channels and narrow beams [47].
- **Low Complex Linear Processing:** More number of base station antenna makes the simple signal detectors and precoders optimal for the system.

## 5. Why Is Massive MIMO Becoming More Important for 5G Networks and beyond?

**Figure 12:**
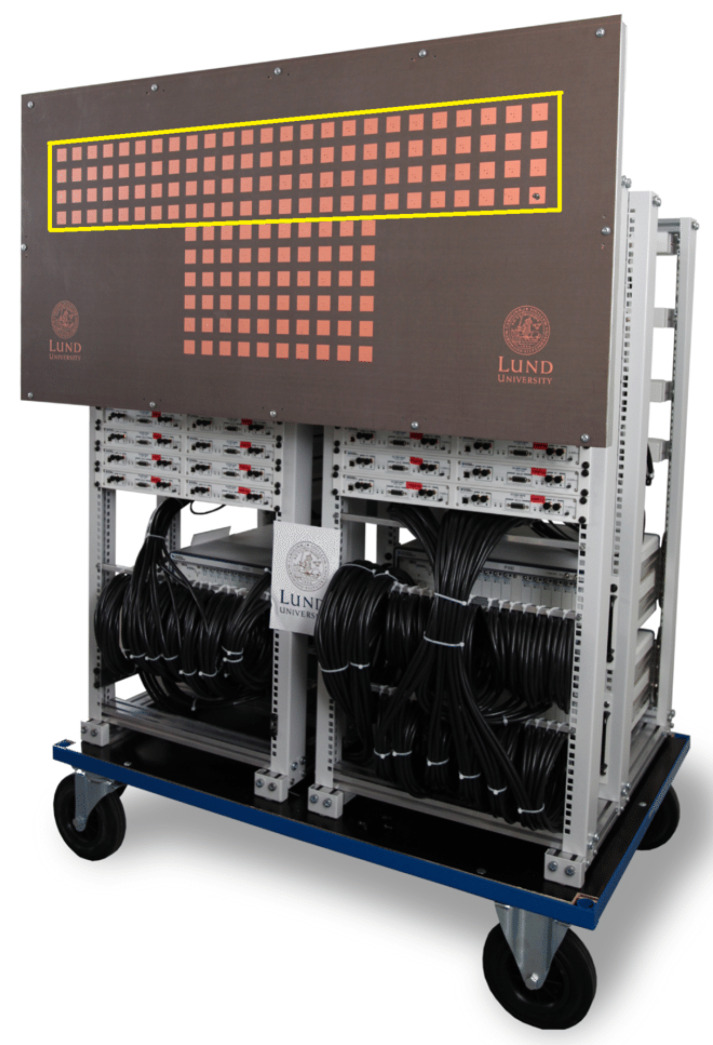
An assembled 100-antenna massive MIMO test bed.

Since the Massive MIMO concept was introduced a few years ago, it has gained new heights every year. It has become one of the hottest research topics in the wireless communication community due to its immense benefits in 5G standardization. The current MIMO systems have been unable to cope with the massive influx in wireless data traffic. With the introduction of concepts like IoT, machine to machine communication, virtual reality, and augmented reality, the current system is unable to deliver the required spectral efficiency. The recent experiments in the massive MIMO system have proven its worth by showing record spectral efficiency. A research conducted by Lund University together with Bristol University in 2015 achieved 145.6 bits/s/Hz spectral efficiency for 22 users, each modulated with 256-Quadrature Amplitude Modulation (256-QAM), on a shared 20 MHz radio channel at 3.51GHz with 128 antennas at the base station [48,49]. Figure 12 shows the 100 antennae massive MIMO testbed created by Lund University in 2015. The improvement in spectral efficiency was huge when compared with 3 bit/s/Hz, which is International Mobile Telecommunications (IMT) advanced requirement for 4G.

The efficient operation of massive MIMO systems has been validated in various environments, both indoor and outdoor. It has also been proven that the massive MIMO system provides a robust operation with low complexity radio frequency and baseband circuit [50]. The hardware implementation of a massive MIMO system also have been tested successfully, and it was proven that these systems could be built with very low complex and low-cost hardware for both digital baseband and analog RF chains [50]. Moreover, many precoding, detection, scheduling, and equalization algorithms have been designed to reduce cost and power further. All these new innovations and development in massive MIMO promote an attractive deployment of this technology required for 5G and beyond wireless networks.

Massive MIMO has already been implemented in China and Japan within a 4G LTE context. SoftBank Group Corp. in Japan deployed massive MIMO in its network in 2016. In 2017, Vodafone and Huawei together did a real-world experiment to test Massive MIMO systems and achieved a speed of 717 Mbps. In 2018, Nokia produced a lightweight and power-efficient chipset for a massive MIMO antenna design, and it was called ReefShark chipset. This chipset could reduce the massive MIMO antenna size to half, and it has been considered as one of the promising technology for Massive MIMO deployment [51]. Samsung also demonstrated that massive MIMO could provide simultaneous high-speed video streaming without delay in a crowded place by experimenting at a crowded stadium in South Korea [52]. In January 2019, Sprint Mobile completed the world’s first 5G data call using 2.5 GHz and Massive MIMO on 3GPP 5G New Radio commercial Network [53].

Theoretically, Massive MIMO systems can have an infinite number of antennas at the base station. But usually, 64 to 128 have been used practically in massive MIMO base station. Recently, Sprint Network working along with companies like leaders Ericsson, Nokia, and Samsung Electronics have deployed 128 antennas massive MIMO systems (64 antennas to receive signal and 64 antennas to transmit signal). One of the prominent advantages of massive MIMO is that we only need sophisticated hardware at the base station, while the UE can have a single antenna and a simple antenna design. Thus, for massive MIMO higher number of the antenna is only needed at the base station but not at UE. The current smartphones have 2 to 4 antennas. The current smartphones have 2 to 4 antennas, but for massive MIMO, having only one antenna at the UE will suffice.

## 6. Challenges in Massive MIMO and Mitigation Techniques

**Figure 13:**
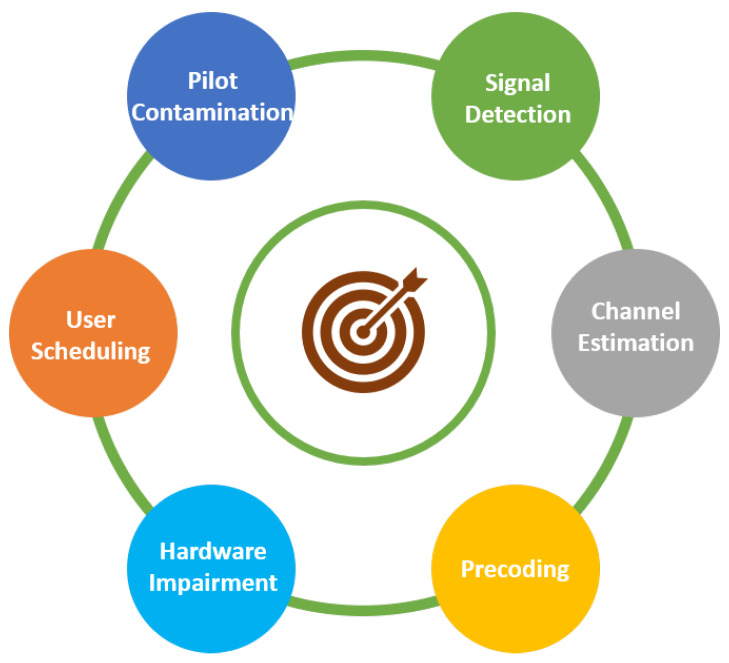
Challenges in massive MIMO deployment.

The massive MIMO technology is more than just an extension of MIMO technology, and to make it a reality, there are still many issues and challenges that need to be addressed. Some of the fundamental challenges in massive MIMO systems are shown in Figure 13.

### 6.1. Pilot Contamination

**Figure 14:**
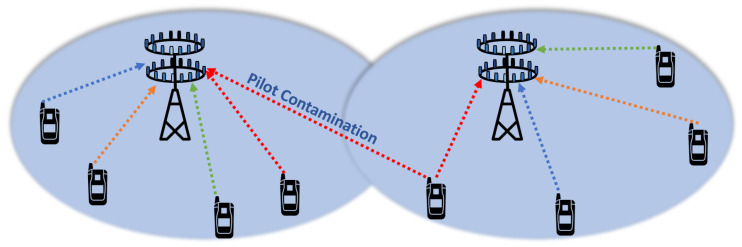
Massive MIMO pilot contamination effect.

In massive MIMO systems, the base station needs the channel response of the user terminal to get the estimate of the channel. The uplink channel is estimated by the base station when the user terminal sends orthogonal pilot signals to the base station. Furthermore, with the help of channel reciprocity property of massive MIMO, the base station estimates the downlink channel towards the user terminal [45]. If the pilot signals in the home cell and neighboring cells are orthogonal, the base station obtains the accurate estimation of the channel. However, the number of orthogonal pilot signals in given bandwidth and period is limited, which forces the reuse of the orthogonal pilots in neighboring cells [54]. The same set of orthogonal pilot used in neighboring cells will interfere with each other, and the base station will receive a linear combination of channel response from the home cell and the neighboring cells. This phenomenon is known as pilot contamination, and it limits achievable throughput, as shown in Figure 14 [55]. During downlink, the base station will beamform towards the user in its home cell along with undesired users in the neighboring cells. The effect of pilot contamination on system performance has been studied in [56,57].

There are several techniques designed to mitigate the effect of pilot contamination in massive MIMO systems. The pilot based estimation approaches are presented in References [58,59]. These pilot based estimation methods show a significant gain when a large number of antennas are used at the base station. The subspace-based estimation approach to mitigate pilot contamination is studied in Reference [60], and it is considered as one of the best methods to increase spectral efficiency because this method required less number of orthogonal pilots. The pilot reuse mitigation scheme is presented in Reference [61], and a partial sounding resource reuse scheme is presented in Reference [62], and these methods are found to be effective in reducing pilot contamination in large antennas systems. A pilot contamination precoding scheme is presented in Reference [63], in which the base station receives the linear combination of signals from all the users using the same orthogonal pilot signal. A blind pilot decontamination method is described in References [64,65] using non-linear receivers. Although blind methods provided accurate channel estimation, its assumption that all the desired channels are stronger than the interfering channel does not always hold [66]. A pilot assignment based scheme and pilot decontamination using interference alignment have been presented in References [67,68]. Some other optimal methods for pilot contamination reduction system designs have been presented in References [69,70]. The author of Reference [71] presented an optimal pilot reuse factor based scheme based upon the user environment to ensure that system always operates at maximal spectral efficiency.

### 6.2. Channel Estimation

**Figure 15:**
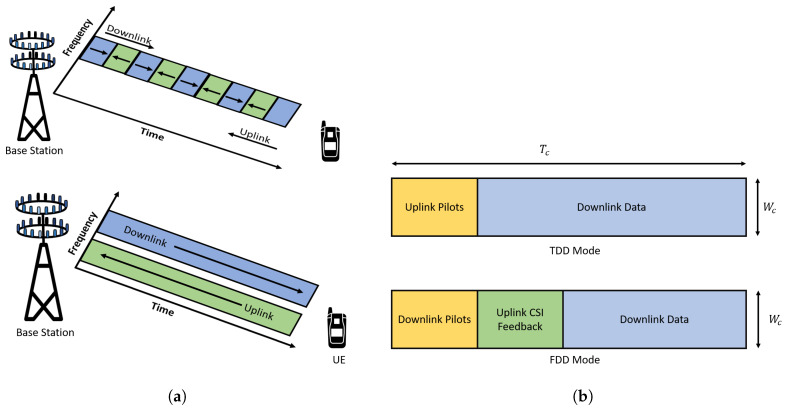
(**a**) Frequency Division Duplexing (FDD) and Time Division Duplexing (TDD) mode: Massive works best in TDD mode. (**b**) Typical pilot transmission and CSI feed back mechanism in FDD and TDD mode.

For signal detection and decoding, massive MIMO relies on Channel State Information (CSI). CSI is the information of the state of the communication link from the transmitter to the receiver and represents the combined effect of fading, scattering, and so forth. If the CSI is perfect, the performance of massive MIMO grows linearly with the number of transmitting or receive antennas, whichever is less [72]. For a system using Frequency Division Duplexing (FDD), CSI needs to be estimated both during downlink and uplink. During uplink, channel estimation is done by the base station with the help of orthogonal pilot signals sent by the user terminal. And during the downlink, the base station sends pilot signals towards the user, and the user acknowledges with the estimated channel information for the downlink transmission. For a massive MIMO system with many antennas, the downlink channel estimation strategy in FDD becomes very complex and infeasible to implement in real-world applications. Figure 15a shows the FDD and Time Division Duplexing (TDD) mode in wireless communication, and Figure 15b shows the typical pilot transmission and CSI feedback mechanism in FDD and TDD mode.

TDD provides the solution for the problem during downlink transmission in FDD systems. In TDD, by exploiting the channel reciprocity property, the base station can estimate the downlink channel with the help of channel information during uplink. During uplink, the user will send the orthogonal pilot signals towards the base station, and based on these pilot signals, the base station will estimate the CSI to the user terminal [54]. Then, using the estimated CSI, the base station will beamform downlink data towards the user terminal. Since there is a limited number of orthogonal pilots that can be reused from one cell to another, the pilot contamination problem arises and is a significant challenge during massive MIMO channel estimation. Other challenges are increased hardware and computational complexity due to more number of antennas. Thus, low complexity and low overhead channel estimation algorithm are very desirable for massive MIMO systems [73].

Recently many algorithms have been designed for channel estimation in massive MIMO systems. A low complex Least Square (LS) estimation is presented in Reference [74], but the accuracy of the method is not optimal. Linear Minimum Mean Square Error (MMSE) algorithm is proposed in References [75,76] and several improvements of the MMSE algorithm are discussed in References [77,78]. Although MMSE provides optimal accuracy, the computational complexity is increased with more number of antennas. The complexity increases due to the large matrix inversion required by the algorithm. The channel estimation based on deep neural networks is presented in Reference [79], which eliminates pilot contamination under certain conditions. The blind channel estimation method is proposed in References [80,81], which are based on subspace properties of the received signal. Compressed Sensing (CS) based channel estimation is proposed in References [82,83], which further improves the downlink channel estimation. Massive MIMO iterative channel estimation and decoding is presented in Reference [84] to improve the complexity performance. Several other optimal methods have been presented recently to address the issue of channel estimation is massive MIMO [85,86,87,88,89,90]. Although massive MIMO is envisioned to use TDD operation, much research has been going on to use FDD operations in massive MIMO systems.

### 6.3. Precoding

Precoding is a concept of beamforming which supports the multi-stream transmission in multi-antenna systems. Precoding plays an imperative role in massive MIMO systems as it can mitigate the effect created by path loss and interference, and maximizes the throughput. In massive MIMO systems, the base station estimates the CSI with the help of uplink pilot signals or feedback sent by the user terminal. The received CSI at the base station is not uncontrollable and not perfect due to several environmental factors on the wireless channel [91]. Although the base station does not receive perfect CSI, still the downlink performance of the base station largely depends upon the estimated CSI.

**Figure 16:**
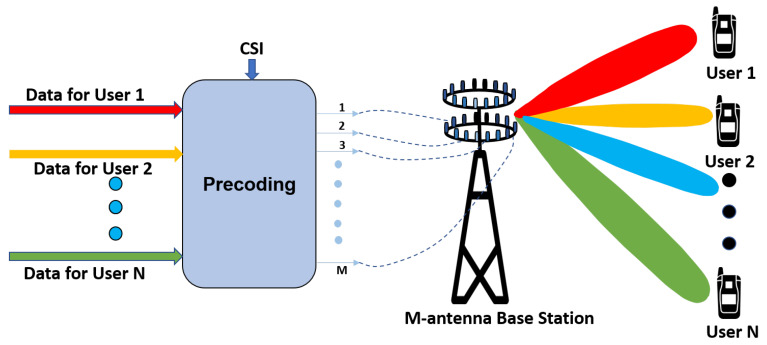
Precoding in a massive MIMO system with M antennas at base station communicating with N users.

Thus, the base station uses the estimated CSI and the precoding technique to reduce the interference and achieve gains in spectral efficiency. The performance of downlink massive MIMO depends upon the accurate estimation of CSI and the precoding technique employed. Although the precoding technique provides immense benefits to massive MIMO systems, it also increases the computational complexity of the overall system by adding extra computations. The computational complexity increases along with the number of antennas. Thus, low complex and efficient precoders are more practical to use for massive MIMO systems. Figure 16 shows the precoding in massive MIMO systems with M-antenna base station and N-users.

Many linear and non-linear precoders have been proposed for massive MIMO systems. Although the non-linear precoders like Dirty Paper Precoding (DPP) [92], Tomlinson Harashima precoding (TH) [93,94], and Vector Perturbation (VP) [95] provide better performance, these methods have very high computational complexity when we have large antenna system. The linear precoders such as Maximal Ratio Combining (MRC) [96], Zero-Forcing (ZF) [97,98], Regularized ZF (R-ZF) [99], Water Filling (WF) [100], and MMSE [101,102] have lower computational complexity and can achieve near-optimal performance.

### 6.4. User Scheduling

**Figure 17:**
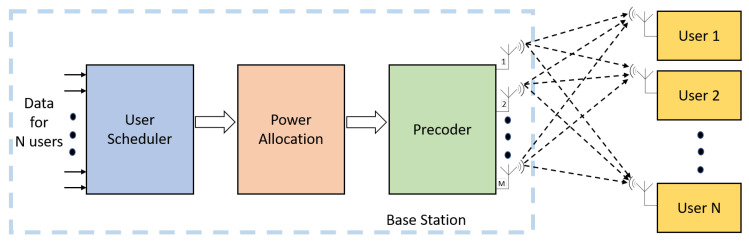
Massive MIMO user scheduling.

Massive MIMO equipped with a large number of antennas at the base station can communicate with multiple users simultaneously. Simultaneous communication with multiple users creates multi-user interference and degrades the throughput performance. Precoding methods are applied during the downlink to reduce the effect of multi-user interference, as shown in Figure 17. Since the number of antennas is limited in massive MIMO base station, if the number of users becomes more than the number of antennas, proper user scheduling scheme is applied before precoding to achieve higher throughput and sum rate performance.

There have been numerous studies in the last few years to find an optimal scheduling algorithm for massive MIMO [103,104]. Several linear methods such as ZF and MMSE provide near-optimal throughput performance and have acceptable computational complexity [105,106]. The non-linear methods such as Dirty Paper Coding (DPC) and Maximum Likelihood (ML) provide near-optimal performance, but they have higher computational complexity for a large number of antenna [92]. Several user scheduling algorithms have been proposed to improve the sum capacity, but computational complexity was not improved for a large number of antennas [107,108]. The Round-Robin (RR) [109], Proportional Fair (PF) [110], and Greedy algorithm [111] guarantee fairness among user. Still, they do not provide optimal throughput performance for massive MIMO systems with a large number of antennas. Multi-user scheduling and joining user scheduling methods have been proposed recently to provide optimal scheduling in a massive MIMO downlink system [112,113]. Several other efficient scheduling methods are proposed in [114,115].

### 6.5. Hardware Impairments

**Figure 18:**
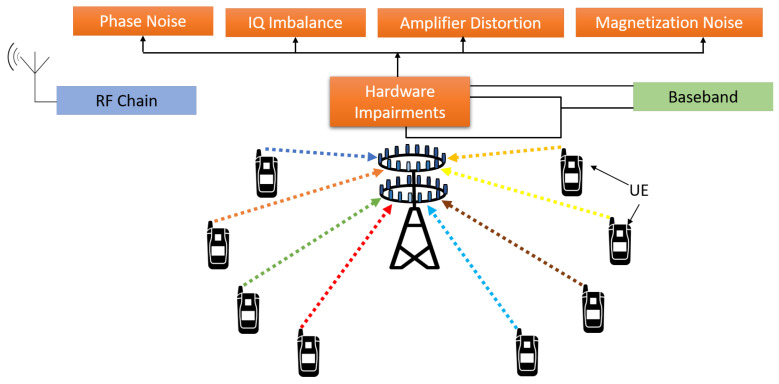
Massive MIMO hardware impairments.

Massive MIMO system depends upon a large number of antennas to reduce the effect of noise, fading, and interference. A large number of antennas in massive MIMO increases the system complexity and increases the hardware cost. To deploy massive MIMO, it should be built with low cost and small components to reduce the computational complexity and hardware size. The use of a low-cost component will increase the hardware imperfections such as phase noise, magnetization noise, amplifier distortion, and IQ imbalance [116]. These imperfections have a major impact on overall system performance. Due to a large number of antennas, there is a mutual coupling between the antenna elements, which changes the load impedance and causes distortions [117]. Although massive MIMO promises to reduces the radiated power 100 times than of conventional MIMO systems, the power consumption by baseband hardware and data converters increases linearly with an increase in the number of antennas. Using low-cost phase-locked loop (PLL) and oscillators increases the phase shift between the time at which pilot and data signal is received at each antenna, which also limits the massive MIMO performance [6]. The hardware impairment at a massive MIMO base station is shown in Figure 18.

Although the hardware impairment cannot to completely removed, its influence can be mitigated with proper use of compensation algorithms. The use of hardware impairment algorithms like phase noise estimation and compensation and digital pre-distortion are infeasible with a large number of antennas, as computational complexity increases exponentially [118,119]. The phase shift problem can be significantly reduced by the design of smart transmission physical layer schemes. To reduce the cost of baseband signal processing, it is highly desirable to build dedicated hardware, which can also run in parallel. The impact of a low-cost amplifier on the transmitter can be mitigated by having a low Peak to Average Power Ratio (PAPR) [120].

### 6.6. Energy Efficiency

Energy efficiency is the ratio of spectral efficiency and the transmit power, and massive MIMO can provide substantial energy efficiency gains by achieving higher spectral efficiency with low power consumption. However, the increasing number of the antenna does always increase the spectral efficiency, because the power consumption also increases along with the number of antenna and more number of users. Based on this analogy, many studies have been carried out to build energy-efficient massive MIMO systems. Many low complex and low-cost methods for precoding, detection, channel estimation, and, user scheduling have been proposed recently to reduce the power consumption at the massive MIMO base station. Some researchers have focused on antenna and power amplifier design to reduce the power consumption of the system. In Reference [121], the authors proposed methods to reduce the mutual coupling induced distortion, but these methods are computationally inefficient for massive MIMO systems.

### 6.7. Signal Detection

**Figure 19:**
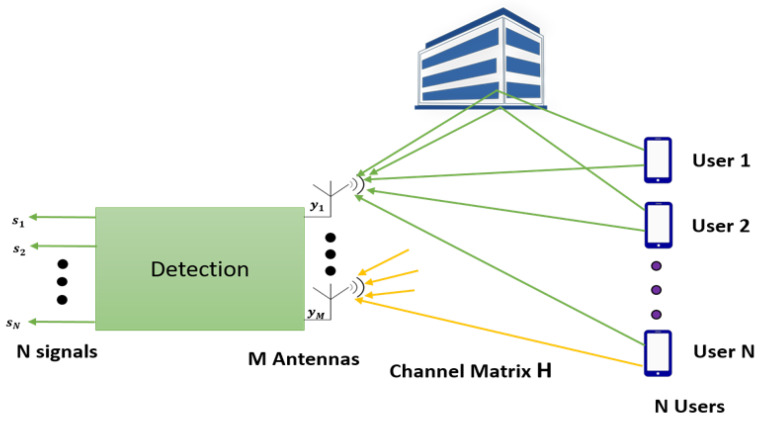
An uplink massive MIMO system.

In massive MIMO systems, due to a large number of antennas, the uplink signal detection becomes computationally complex and reduces the achievable throughput. Also, all the signals transmitted by users superimpose at the base station to create interference, which also contributes to the reduction of throughput and spectral efficiency. Figure 19 shows a massive MIMO system with N user terminal and M antenna at the base station. All the signals transmitted by N user terminal travel through a different wireless path and superimpose at the base station, which makes signal detection at the base station complex and inefficient. There has been extensive research to find the optimal signal detection method for massive MIMO systems that can provide better throughput performance with lower computational complexity. The conventional non-linear detectors like Sphere Decoder (SD) [122] and Successive Interference Cancellation (SIC) [123] yield good performance. Still, the computational complexity increases with more number of antennas, which makes them infeasible for massive MIMO systems.

Several linear detectors have been considered for uplink detection in massive MIMO, such as ML, ZF, and MMSE [47,124]. ML is an optimal detector in massive MIMO, and it minimizes the probability of error, but for large antennas systems, the algorithm has prohibitive complexity [125,126]. The ZF methods mitigate the inter-antenna interference, but for ill-conditioned channel matrices, additive noise gets increased [127]. The MMSE detector has better performance than the ZF detector as it also considers the noise power during the detection [128]. Although the ML, MMSE, and ZF detection algorithms provide optimal throughput performance, they involve matrix inversion during the processing, which makes them computationally inefficient for large antenna massive MIMO systems. The ZF and MMSE algorithms combined with the Successive Interference Cancellation (SIC) method were considered to cancel the interference from previously detected symbols [129]. For low complexity signal detection of massive MIMO systems, several iterative methods have been designed [130,131]. Neumann Series Approximation (NSA) method [132], Richardson method [133], Successive Over-Relaxation Method (SOR) [74], and Jacobi Iterative Method [134] have been considered, but computational complexity was slightly reduced, when compared to conventional linear methods. Other linear methods such as Gauss Siedel (GS) [135], Conjugate Gradient (CG) [131], Least-square regression selection [136], Huber fitting based Alternating Direction Method of Multipliers (ADMM) [137], and Approximate Message Passing (AMP) [138] methods were also considered for massive MIMO, but they were also not found optimal for massive MIMO uplink detection. Several other optimal algorithms for massive MIMO uplink signal detection are presented in References [86,139,140,141,142,143].

## 7. Can Our Current Mobile Phones Use Massive MIMO Technology?

Our current phones do not support massive MIMO systems, and you cannot buy a massive MIMO ready phone yet. Even if you buy a phone which supports massive MIMO, it will not be beneficial until we have massive MIMO supporting wireless network. However, many phones now benefit from MIMO technology to achieve higher data rates and reliability. Every antenna embedded on the phone is used for transmitting and receiving the data. The added number of the antenna means, your device can send and receive more data at once. Hence this will boost the upload and download speeds. Today, most of the flagship phones come up with 4 × 4 MIMO, and they are two times faster than the phones having 2 × 2 MIMO as they will have two free antennas. Currently, iPhone XR, iPhone X, and iPhone 11 are equipped with 2 × 2 MIMO whereas iPhone 11 pro, iPhone 11 pro-Max, iPhone XS Max, Samsung Galaxy S8/S9/S10, Google Pixel 2/Pixel 3, HTC U11/U12+, and Huawei Mate 20 Pro are some of the phones that support 4 × 4 MIMO [144]. Although your phone does not support the massive MIMO system, you can still get benefit from the massive system as the connection would be more reliable and sensitive. Overall, the reliable connection and higher data are always good to have, but you have to pay some extra bucks to use massive MIMO technology.

## 8. Machine Learning and Deep Learning for Massive MIMO Systems

Machine learning is a subset of artificial intelligence, which is known as a powerful tool for classification and prediction problems. Deep learning is a subset of machine learning, and it uses more advanced tools capable of building universal classifiers and approximate general functions. These new concepts have been widely used in areas such as natural language processing, network security, and automated systems (autonomous cars). Currently, both machine learning and deep learning are very crucial technology for the design of 5G and 6G networks. Massive MIMO requires very complex optimizations, and the traditional algorithm, such as stochastic geometry and game theory, are very sophisticated and require enormous computing power. The dynamic nature of machine learning and deep learning algorithms could be instrumental for there complex analysis, and it could save a considerable amount of computational power [145]. These machine learning and deep learning algorithms are useful during massive MIMO beamforming, channel estimation, signal detection, load balancing, and optimization of available spectrum [146,147]. The uses of deep learning and machine learning for massive MIMO have been studied in [145,148].

**Figure 20:**
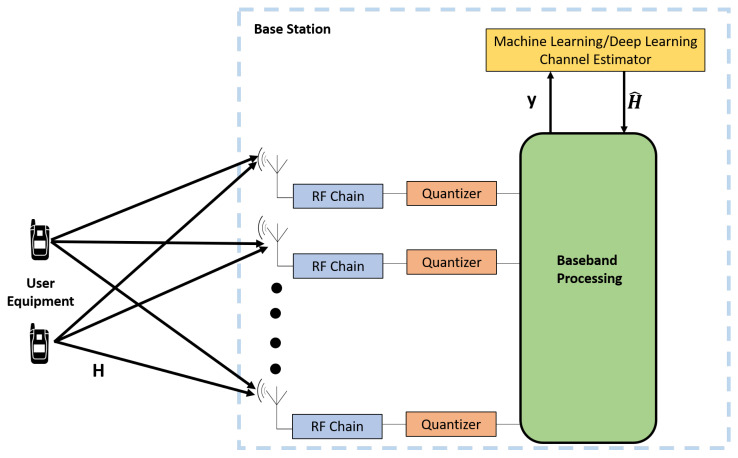
Massive MIMO channel estimation using machine learning and deep learning.

During channel estimation, channel data can be considered as big data, and several machine learning tools can be used to predict massive MIMO channels. The accurate prediction of the channel via machine learning with significantly improve the throughput of massive MIMO systems. The use of machine learning or deep learning for channel estimation in massive MIMO is shown in Figure 20. The authors of Reference [149] have used the Convolutional neural network (CNN) method for channel estimation, but the optimal performance was not achieved. CNN combined with a projected gradient descent algorithm was presented in Reference [150] that demonstrates the feasibility of using machine learning methods in channel estimation. The use of machine learning to estimate channel in complex channel model conditions has been studied in Reference [151]. Deep learning-based channel estimation has predicted more accurate channels compared to conventional channel estimation algorithms [152]. The authors of Reference [153] considered a massive MIMO channel as an image and applied a deep learning image super-position and denoising method. Various other research has been conducted to develop end to end Deep neural network (DNN) architecture to modify the modules at the base station and UE’s [154]. Deep learning-based channel estimation for various scenarios have been presented in Reference [155], and the results were like those of the optimal MMSE algorithm. Machine learning algorithms can reduce channel estimation overhead during CSI estimation in massive MIMO systems. Deep learning-based sparse channel estimation methods and their advantages over traditional estimation methods have been presented in Reference [90]. The CSI estimation problem in massive MIMO can be considered as time series learning problem by considering channel aging property of massive MIMO. The recurrent neural network (RNN) is a powerful tool to solve this time series learning problem. Since CSI estimation has distant data, simple RNN tools are less efficient in predicting the distant data in wireless communication. Thus, several architectures have been proposed recently to address this distant data problem in massive MIMO, such as long short-term memory (LSTM) and non-linear autoregressive network with exogenous inputs (NARX) [156,157]. Machine learning-based channel prediction in a massive MIMO system with channel aging property has been studied in Reference [158].

CNN combined with the autoregressive network (ARN), and RNN has been studied in Reference [158]. The machine learning assisted user scheduling method presented in Reference [159] provides a low complexity scheduling scheme for massive MIMO systems. The authors of Reference [160] presented a novel channel mapping in space and frequency using deep learning in massive MIMO. This novel solution reduces the training and feedback overhead in massive MIMO systems. Machine learning has also been used for efficient beam alignment in massive MIMO systems to track the users efficiently [161]. Several machine learning and deep learning techniques are also useful for uplink signal detection in massive MIMO. The conventional signal detection methods are computationally very complex and inefficient for large antennas systems like massive MIMO. Several semi-supervised learning (SSL) [162] and supervised learning (SL) [163] approach have been proposed and provide more robust performance. Several other uses of machine learning and deep learning have been presented in Reference [164,165,166,167].

## 9. Active Research Topics on Massive MIMO for 5G and beyond Networks

Although massive MIMO provides immense benefits, there are still various challenges such as pilot contamination, channel estimation, precoding, user scheduling, hardware impairments, energy efficiency, and signal detection that needs to be addressed and tested in a real-world environment before we can achieve its promised advantages. These deployment challenges in massive MIMO systems have pushed both academia and industry to focus on massive MIMO systems. Also, new technologies like massive MIMO, ultra massive MIMO, millimeter waves, terahertz waves, and visible light communication needs a lot of research before it gets implemented in our current wireless system. Some of the possible research topics in massive MIMO for 5G and beyond networks are:

- Massive MIMO system depends upon a large number of antennas to reduce the effect of noise, fading, and interference. A large number of antennas in massive MIMO increases the system complexity and increases the hardware cost. To deploy massive MIMO, it should be built with low cost and small components to reduce the computational complexity and hardware size. The low-cost equipment will increase the hardware imperfections such as phase noise, magnetization noise, amplifier distortion, and IQ imbalance. Although the hardware impairment cannot to completely removed, its influence can be mitigated with proper use of compensation algorithms. Design of these compensation algorithms is a good area of research in massive MIMO.
- Since there are limit number of orthogonal pilots that can be used in a particular time, the pilot contamination becomes one of the significant challenges in massive MIMO deployment. Pilot contamination increases interference and limits the achievable throughput. Several research has been conducted to mitigate the effect of pilot contamination. However, there is a need for an optimal method that mitigates its effect [58,59,60,61,62,63,64,65,66,67,68,69,70]. Thus, effective ways to mitigate the pilot contamination effect is an essential area to investigate.
- Although the precoding techniques increase throughput and reduce interference, it increases the computational complexity of the overall system by adding extra computations. This computational complexity increases with a large number of antennas. Thus, it is more practical to use low complex and efficient precoders in massive MIMO. Through investigation to find efficient precoding technique for massive MIMO is also an essential area of research.
- Since there are a limited number of antennas in the massive MIMO base station, user scheduling has to be performed if the number of the users is more than the number of antenna terminals at the base station. Massive MIMO system throughput can be increased by only scheduling the users experiencing good channel conditions. But using this scheme, the users at the edge of the cell with poor channel conditions are ignored and never scheduled. To improve overall system performance, a certain amount of fairness must be ensured among all the users. Several research has been conducted to achieve an efficient user scheduling algorithm [92,105,106,107,108,109,110,111,112,113,114,115], but optimal performance has not been achieved. Further research should be conducted to find a more efficient and fair scheduling algorithm design that can provide a higher data rate and guarantee fairness among users.
- In massive MIMO systems, due to a large number of antennas, the uplink signal detection becomes computationally complex and reduces the achievable throughput. Also, all the signals transmitted by users superimpose at the base station to create interference, which also contributes to the reduction of throughput and spectral efficiency. A recent experiment has achieved near-optimal performance, but more efficient algorithms are required to realize massive MIMO [47,74,86,122,123,124,125,126,127,128,129,130,131,132,133,134,135,136,137,138,139,140,141,142,143]. One of the crucial areas of investigation is to find more efficient and low complex uplink signal detection algorithm.
- Accurate CSI is needed in massive MIMO for beamforming data, detecting user signal, and resource allocation [168]. The user terminal has to estimate signal coming from a large number of antennas at the base station. Furthermore, the pilot overhead also increases drastically. Thus, an efficient channel estimation scheme with reasonable pilot overhead is an exciting area to investigate, particularly for FDD scheme.
- An exciting area for research in massive MIMO will be to combine it with quantum communication with a frequency higher than 300 GHz.
- Massive MIMO technology will be used for a user having a large number of antennas. Massive MIMO transceiver design, complexity, performance should be tested with users having a large number of antennas.
- Since the phones available in the current market does not support massive MIMO infrastructure; it would be a challenge for device manufacturers to develop cheaper phone which can support this technology. Design of a massive MIMO system that can integrate with the current 4G network is an excellent area to study.
- The use of machine learning and deep learning algorithms during massive MIMO channel estimation to predict statistical channel characteristics is an exciting area of research. Several experiments have been conducted recently to explore machine learning and deep learning for massive MIMO channel estimation, user scheduling, beamforming, and signal detection [90,149,150,151,152,153,154,155,156,157,158,159,160,161,162,163,164,165,166,167].
- The study on potential key enabling technologies for 6G networks such as THz communication, visible light communication, and holographic radio is also an interesting area to investigate.
- Further investigation is required to realize THz UM-MIMO for 5G and beyond networks. Some of the areas to the important area to investigate are the fabrication of plasmonic nano array antennas, optimal channel estimation methods, low complex and efficient precoding, and signal detection algorithms, accurate beamforming, and beemsteering [16,17].

Table 3 provides a summary of the massive MIMO system, its characteristics, benefits, and challenges. Table 4 summarizes the fundamental challenges in massive MIMO system implementation and recently proposed mitigation techniques.

## 10. Conclusions

The need for an efficient cellular spectrum that can accommodate the tremendous surge in wireless data traffic is imminent. Massive MIMO wireless access technology is the answer to this global demand. Massive MIMO technology groups together antennas at both transmitter and the receiver to provide high spectral and energy efficiency using relatively simple processing. Given the worldwide need for an efficient spectrum, a limited amount of research has been conducted on massive MIMO technology. Thus, several open research challenges are still in the way of this emerging wireless access technology.

This paper provides an extensive overview of massive MIMO systems, highlighting the key enabling technologies for 5G and beyond networks. Although massive MIMO offers immense benefits for 5G and 6G networks, there are still various deployment challenges such as pilot contamination, channel estimation, precoding, user scheduling, hardware impairments, energy efficiency, and signal detection that needs to be addressed before we can achieve its promised advantages. Furthermore, this paper outlines the recent trends such as terahertz communication, UM-MIMO, VLC, and application of machine learning and deep learning technology for massive MIMO systems. We hope that this paper will motivate the researchers currently working on 5G and beyond networks field to find new paths and open problems to tackle in the coming years.

## Figures and Tables

**Table 1:** Features of 6G Networks.

Performance Index	4G	5G	6G
Peak Data Rate	100 Mbps	10 Gbps	Upto 10 Tbps
Latency	10 ms	1 ms	Upto 0.1 ms
Connection Density	0.1 million devices/km${}^{2}$	1 million devices/km${}^{2}$	10 million devices/km${}^{2}$
Energy Efficiency	1×	100 × 4G	100 × 5G
Spectral Efficiency	1×	100 × 4G	100 × 5G
Available Spectrum	Upto 6 GHz	Upto 300 GHz	Upto 3 THz
Mobility	200 m/h	300 m/h	600 m/h
Artificial Intelligence	No	Partial	Fully

**Table 2:** Comparison of Traditional MIMO and Massive MIMO System.

	MIMO	Massive MIMO
Number of Antenna	≤8	≥16
Pilot Contamination	Low	High
Throughput	Low	High
Antenna Coupling	Low	High
Bit Error Rate	High	Low
Noise Resistance	Low	High
Diversity/Capacity Gain	Low	High
Energy Efficiency	Low	High
Cost	Low	High
Complexity	Low	High
Scalability	Low	High
Link Stability	Low	High
Antenna Correlation	Low	High

**Table 3:** Summary of Massive MIMO System, its Characteristics, Benefits, and Challenges.

Feature	Massive MIMO System
Main aspect	Base station with hundreds of antennas
	Multiple users
	Low power antennas
Characteristics	Many more antennas than number of users
	Multiplexing gain
	Small low power antennas
	Very directive signals
	Little interference leakage
Technical Content	Number of antennas ≥ 16
	High channel capacity
	High throughput
	High antenna coupling
	Low BER
	High noise resistance
	High implementation cost
	High scalability
	High link stability
	High antenna correlation
Benefits	High spectral efficiency
	Array gain
	High energy efficiency
	High data rate
	User tracking
	Low power consumption
	Less fading
	Low latency
	More reliability
Challenges	Pilot contamination
	Channel estimation
	Precoding
	User scheduling
	Hardware impairments
	Energy efficiency
	Signal detection

**Table 4:** Summary of Challenges and Mitigation Techniques in Massive MIMO System.

Challenges	Mitigation Techniques
Pilot Contamination	Pilot based Estimation [58,59], Subspace based Estimation [60], Pilot Reuse [61], Partial Sounding Resource [62], Pilot Contamination Precoding [63], Blind Pilot Decontamination [64,65], Pilot Decontamination [69], Distributed Non-Orthogonal Pilot Design [70].
Channel Estimation	Least Square [74], MMSE [75,76], Improved MMSE [77,78], Blind Estimation [80,81], Compresses Sensing [82,83], MICED [84], Untraind Deep Neural Network [85], Compressed Sensing [86], Convolutional Blind Denoising [87], VAMP [88], Deep Learning based Sparse Estimation [89], CNN based Estimation [150], Machine Learning based Estimate [151,158], Deep Learning based Estimation [153,155]
Precoding	DPP [93], TH [94,95], VP [96], MRC [97], ZF [98,99], WF [100], MMSE [101,102]
User Scheduling	ZF [105], MMSE [106], DPC [92], RR [107], PF [108], Greedy [109], Multi-user Grouping [112], Gibbs Distribution Scheme [114], Pilot Efficient Scheduling [115], Machine Learning based Scheduling [159]
Hardware Impairments	Digital Pre-Distortion [118,119], PAPR [120],
Signal Detection	SD [122], SIC [123], ML [47], ZF [124], MMSE [125], NSA [132], Richardson [133], SOR [74], Jacobi [134], Gauss Siedel [135], Conjugate Gradient [131], Least Square Regression Selection [136], Huber ADMM [137], AMP [138] Compressed Sensing based Adaptive Scheme [86], CNN [140], Gauss Siedel Refinement [143], SSL and SL based Detection [162,163], APRGS [169]

## Abbreviations

The following abbreviations are used in this manuscript:

MIMO	Multiple-input multiple-output
IoT	Internet of things
M2M	Machine to machine
LTE	Long term evolution
LAN	Local area network
MAC	Media access control
FDMA	Frequency division multiple access
AMPS	Advanced mobile phone systems
TACS	Total access communication system
TDMA	Time division multiple access
CDMA	Code division multiple access
3GPP	3rd Generation Partnership Project
GSM	Global system for mobile communication
GPRS	General packet radio service
EDGE	Enhanced data GSM evolution
MMS	Multimedia message support
HSPA+	High speed packet access
HSDPA	High speed downlink packet access
HSUPA	High speed uplink packet access
QoS	Quality of service
HDTV	High definition television
WiMAX	Worldwide interoperability for microwave access
QAM	Quadrature amplitude modulation
IMT	International mobile telecommunications
CSI	Channel state information
CS	Compressed sensing
FDD	Frequency division duplexing
TDD	Time division duplexing
LS	Lease square
MMSE	Minimum mean square error
DPP	Dirty paper precoding
TH	Tomlinson Harashima
VP	Vector perturbation
MRC	Maximal ratio combining
ZF	Zero-Forcing
R-ZF	Regularized zero-forcing
WF	Water filling
RR	Round robin
PF	Proportional fair
PLL	Phase-locked loop
PAPR	Peak to average power ratio
SD	Sphere decoder
SIC	Successive interference cancellation
NSA	Neumann series approximation
SOR	Successive over-relaxation
ADMM	Alternating direction method of multipliers
AMP	Approximate message passing
DL	Deep learning
CNN	Convolutional neural networks
RNN	Recurrent neural networks
DNN	Deep neural networks
LSTM	Long short-term memory
NARX	Nonlinear autoregressive network with exogenous inputs
ARN	Autoregressive network
SSL	Semi-supervised learning
SL	Supervised learning
MICED	MIMO iterative channel estimation and decoding
VAMP	Variational approximate message passing
APRGS	Accelerated and Preconditioned Refinement of Gauss-Seidel
UM-MIMO	Ultra massive MIMO
SU-MIMO	Single user MIMO
MU-MIMO	Multi user MIMO
VLC	Visible light communication
